# Self-Assessment of Health Professionals’ Cultural Competence: Knowledge, Skills, and Mental Health Concepts for Optimal Health Care

**DOI:** 10.3390/ijerph191811282

**Published:** 2022-09-08

**Authors:** Alexandros Argyriadis, Evridiki Patelarou, Panagiotis Paoullis, Athina Patelarou, Ioannis Dimitrakopoulos, Vasiliki Zisi, Ruth Northway, Maritsa Gourni, Evanthia Asimakopoulou, Dimitra Katsarou, Agathi Argyriadi

**Affiliations:** 1 School of Health Sciences, Frederick University, Nicosia 1036, Cyprus; 2 School of Health Sciences, Hellenic Mediterranean University, 714 10 Iraklio, Greece; 3 Department of Physical Education and Sport Science, University of Thessaly, 382 21 Volos, Greece; 4 Faculty of Life Sciences and Education, University of South Wales, Pontypridd CF37 1DL, UK; 5 Department of Pre-School Education and Educational Design, University of the Aegean, 811 00 Mitilini, Greece; 6 School of Education and Social Sciences, Frederick University, Limassol 3080, Cyprus

**Keywords:** cultural competence, personalized care, self-assessment, quality health care, optimal care, mental health, diversity

## Abstract

Current research often refers to cultural competence to improve health care delivery. In addition, it focuses on the cultural uniqueness of each health service user for optimal personalized care. This study aimed to collect self-assessment data from health professionals regarding their cultural competence and to identify their development needs. A mixed methods design was adopted using the Cultural Competence Self-assessment Checklist of the Central Vancouver Island Multicultural Society. This was translated into Greek, validated, and then shared with health professionals in Cyprus. Subsequently, a semi-structured interview guide was designed and utilized. This was structured in exactly the same question categories as the questionnaire. Data collection took place between October 2021 and May 2022, and convenience sampling was used to recruit 499 health scientists in Cyprus. The sample comprised doctors, nurses, psychologists, midwives, social workers, and physiotherapists. Subsequently, 62 interviews were conducted with participants from the same specialties. The results showed that (compared to other health professionals) nurses and psychologists are more sensitive to issues of cultural competence. It would appear that the more socially oriented sciences had better-prepared healthcare staff to manage diversity in context. However, there is a gap between knowledge and skills when comparing doctors to nurses; they seem to be more skilled and willing to intervene actively in cases of racist behavior or problem-solving. In conclusion, participants identified the importance of their cultural competence; they also realized the importance of optimal planning of personalized health care. There is a significant need for continuous and specialized cultural competence training for all health professions.

## 1. Introduction

Research that aims to enhance the quality of personalized health care delivery has recently focused on the cultural competence of health professionals. Cultural competence is referred to as the process by which the health care provider constantly strives to work effectively according to the patient’s cultural context [1]. This ability includes characteristics such as respect, adapting care to the values, needs, practices, and expectations of individuals, as well as providing fair and ethical care and understanding [2]. The characteristics of cultural competence, according to Zarzycka et al., are cultural awareness, knowledge, sensitivity, skills, competence, and dynamics [3]. These variables affect the health literacy of members of the same ethnic group, health behaviors, perceived risk, attitudes, and beliefs towards health care [4]. Therefore, several studies argue that to ensure effective and culturally adequate care and to develop culturally competent health care practices, properly educated providers are required [3,5]. These practices are directly related to the mental health characteristics of professionals necessary for effective cooperation between those involved in health services. In this context, cultural competence assessment tools, such as the “Cultural Self-Efficacy Scale (CSES),” for the self-efficacy of nurses caring for specific groups of people, including colored, Hispanic, and Asian patients, have been developed [6]. The Cultural Competence Assessment Scale-CCAS assesses the cultural awareness, sensitivity, and behavior of physicians [7], Self-Efficacy Scale-CSES, assesses cultural knowledge and skills [8]. Other tools that have been used successfully are the Transcultural Assessment of Self-Efficacy Scale, which measures cultural awareness, knowledge, and skills; the Cultural Capacity Scale-CCS, for the assessment of cultural sensitivity, knowledge, and skills; Nurses ‘Cultural Competence Scale-NCCS; the Scale of Cultural Competence for nurses-SCC; and Scale of Community Health Nurses’ Cultural Sensitivity-SCS [1,9].

Recent research and the tools used, however, show a significant lack of using mixed methods and self-assessment scales. In particular, in Cyprus, there are no recent systematic reviews regarding cultural competence in the healthcare sector, nor has this issue been studied at a national level. In Greece and Cyprus, the importance of this has not been prioritized, nor have specific training programs been implemented appropriately and in accordance with the local needs of the communities. This is a particular issue within Cyprus as the geographical position of the country and the financial opportunities that it offers attract people from Europe, Asia, and Africa, making it more multicultural. In addition, the population heterogeneity of Cyprus includes (apart from refugee minorities) every other community within the spectrum of diversity and has its own cultural identity. At the same time, there is an evident lack of governmental, educational, and social emphasis on cultural competence, knowledge, and skills [10]. There are also no data related to intervention programs to promote cultural competence, nor are there relevant topics in the curricula of medical and nursing schools. The lack of cultural capacity in health professionals, constant social changes, and the intense development of multiculturalism leads to the growing and intense inequality and marginalization of minorities or vulnerable groups receiving quality and effective health care services [11,12]. In addition, the number of systematic international reviews is low, and there are several studies that focus primarily on nurses but not other health professionals. Thus, the rights of vulnerable communities, diversity, multiculturalism, culture, beliefs, values, attitudes, views, experiences, patterns of thought, rules of conduct, and forms of communication are often overlooked. However, they continue, are transmitted from generation to generation, and are subject to change and evolution [13]. As a result, there is a reduced quality in the personalized provision of health services [14].

## 2. Materials and Methods

This study aimed to measure the level of knowledge and skills of cultural competence that the participants consider themselves to possess through the collection of self-assessment cultural competence data from health professionals. For this reason, this study used mixed methods, combining the quantitative and qualitative methodology.

The Cultural Competence Self-assessment Checklist of the Central Vancouver Island Multicultural Society was used to collect data after it had been translated into the Greek language and validated [10]. Next, a semi-structured interview guide was designed for the study, structured in exactly the same categories as the questionnaire but using different questions. In this way, it was possible to cross-check the answers and collect additional data as well as non-verbal information.

The following research questions emerged from the review of the literature and the identification of gaps in the research:

1. How do health professionals evaluate their knowledge of transcultural communication?
2. How do health professionals perceive their transcultural communication skills?
3. What is the relationship between the self-assessment of health professionals and the analysis of their qualitative interview responses?

**Data Collection and Sample of the Study**

Data collection took place between October 2021 and May 2022 from 499 health scientists who live and work in Cyprus. Convenience sampling was used. The Northern part of Cyprus (which is occupied by Turkey and is not recognized internationally as an independent country) was excluded from this study due to access difficulties. The sample comprised doctors, nurses, psychologists, midwives, social workers, and physiotherapists. Subsequently, 62 interviews were conducted with participants from the same specialties (Table 2).

**The questionnaire**

The Self-Assessment tool was developed by the Central Vancouver Island Multicultural Society with the ultimate goal of helping people to consider their skills and knowledge in their interactions with others. Its goal was to assist communities in recognizing what they can do to become more effective in working and living in a diverse environment. It comprises a Likert rating scale to help respondents identify areas of strength and areas that need further development to reach cultural competence. The response categories were set as Never/Not at all, Sometimes/Good, Often/Fairly good, and Always/Excellent, with the score being respectively totaled at the end of each section. The questions used deal with cultural knowledge, about learning from mistakes, the assessment of the knowledge, the questions an individual asks themself in terms of cultural difference, and the importance that this difference has to the individual. It also includes questions about knowledge of history, understanding the impact of culture, interest in lifelong learning, and understanding the consequences of racism, sexism, homophobia, etc. Finally, this section includes questions about the participant’s knowledge of the history of diversity. The section listing skills has questions about adaptability to diversity, active support for people on the diversity spectrum, and transcultural communication skills. It also records the search for opportunities to acquire skills and the active involvement of the individual in processes that promote cultural experiences. Respect for diversity and the implementation of cultural practices in combination with allied strategies and flexibility are some of the necessary elements that are recorded to develop a more complete picture of the cultural ability of the individual.

**The semi-structured interview guide**

To capture the cultural competence of health professionals more fully, it was also felt necessary to use a qualitative methodology to explore the experiences of the participants from their collaboration with diverse communities. A semi-structured interview guide was designed, which included nine questions grouped into two main categories: (a) questions about how the participants acquired knowledge about cultural diversity and best practices, the management of transcultural communication, previous studies, and the role of the social environment, and (b) questions about experiences of active involvement of health professionals in cases they had to intervene in or deal with.

**The Procedure**

The study was part of the research program entitled: QR-CCC Building Cultural Competence for Health Professionals, funded by Frederick University between September 2021 and June 2022. After the research was designed, the quantitative research instrument was translated into Greek and validated following international guidelines for the validation of research tools published in a reputable journal [10]. It was then applied to the Cypriot health care professional population following an invitation to also participate in the qualitative research. After expressing their interest, the participants were asked to answer the questionnaire in a personal interview. All interviews were conducted by the research team members who were qualified in qualitative research methods. The duration of the quantitative element of the interview was 20 minutes, and this was then followed by the semi-structured interview process, which took another 30 minutes. In all instances, the procedure was performed in a quiet place within the working environment of health professionals. Analysis of quantitative data used the statistical program SPSS v.25 (Chicago, IL, USA). Qualitative data were analyzed using thematic content analysis. Using content analysis, the researchers quantified and analyzed the presence, meanings, and relationships of certain words, themes, or concepts. The researchers were, thus, able to evaluate the language used and to search for bias or partiality. After familiarizing themselves with the data, the researchers coded and generated themes. After reviewing the aforementioned steps, the research team defined theoretical models leading to the writing up and their theoretical analysis.

**Ethical Issues**

The written approval of the Central Vancouver Island Multicultural Society was obtained by the scientific director of the organization for its use in this study. Approval was also obtained from the Frederick University Bioethics Committee, as well as written consent from participants. Throughout the process, anyone was able to withdraw their participation, and everyone was made aware of their rights. No names were recorded on the gathered data and participants were aware that no individual data would be identifiable in any reports/publications.

**Validity and Reliability**

The English language version of the Cultural Competence Self-assessment Checklist of the Central Vancouver Island Multicultural Society has been tested for validity and reliability. The reliability has been tested using Cronbach’s α method. In addition, the internal structure was tested by a confirmatory factor analysis-CFA, and the validity analysis has also been taken into account. The measurements of the Cronbach alpha values were, on average, 0.80, so it is considered to be a reliable tool (Table 1).

## 3. Results

The results of the research are presented in two sections: (a) those obtained from the self-assessment questionnaire for the ten questions that measured the knowledge and ten questions that measured the attitudes of health professionals, and (b) the findings obtained from the semi-structured interviews.

### 3.1. Data Analysis

#### 3.1.1. Results from the Questionnaire

499 health professionals expressed an interest in participation, but due to drop-outs or incomplete completion of the questionnaire, the final participant number was 405 health professionals. So, the participation rate was 81.17% of the initial population. Sixteen point eight per cent were men (n = 68) and 83.2% women (n = 337). Fifty three point eight per cent (n = 218) were nurses, 22% (n = 89) doctors, 9.4% (n = 38) midwives, 9.4% (n = 38) psychologists and 5.4% (n = 22) were other allied health professionals. There were 31.1% (n = 126) of participants who only had an undergraduate degree, while 68.6% (n = 278) also had a postgraduate degree. Only 0.2% (n = 1) of them also had a doctorate. There were 60.2% (n = 244) of participants who stated that they had previous education in cultural studies, while 39.8% (n = 161) did not. Finally, 48.4% (n = 196) of the participants came from urban centers and 51.6% (n = 206) from the provinces (Table 2). It is worth noting that the minimum qualification for every health professional to practice his science in Cyprus is the Bachelor’s degree and any other qualification (for example, MSc or Ph.D.) are additional specializations.

The first questions in the research tool relate to participants’ evaluation of their knowledge, which is a factor that shapes individual cultural competence (see Table 3). More specifically, in the first question about learning from their mistakes, 13.6% (n = 55) answered that they do not make mistakes in their behavior with people from different cultural backgrounds and 31.4% (n = 127) answered that it sometimes happens.

Another question states, “I recognize that my knowledge of certain cultural groups is limited and commit to creating opportunities to learn more.” To this, a high percentage of participants answered always and often with percentages of 50.4% and 40.2%, respectively. Only a small percentage of 1.2% (n = 5) answered that they are not interested in being trained in transcultural communication issues. Women seem to answer more positively compared with men, with a statistically significant difference (*p* = 0.046).

Ninety-five percent (n = 385) of the participants indicated they would listen to the responses before asking another question about people who belong to a diverse community. Moreover, negative answers were found in very low percentages.

There were 49.9% (n = 202) of participants who self-reported an excellent knowledge that differences in color, culture, and ethnicity are important parts of an individual’s identity, which they value. A further 37.8% (n = 153) reported a fairly good knowledge.

In response to the question of whether the participants are knowledgeable about historical incidents in Cyprus that demonstrate racism and exclusion towards them, only 10.6% (n = 43) answered that they have excellent knowledge, 33.3% (n = 135) fairly well, 43.7% (n = 177) moderate and 12.3% (n = 50) not at all. People who had previously attended seminars about cultural issues stated that they are more knowledgeable about their history and the history of others. This difference was statistically significant, *p* = 0.01.

The recognition that cultures change over time can vary from person to person, as does attachment to culture, and the majority of the sample answered positively with a rate of 89.6% (n = 363).

Moreover, 93.1% (n = 377) of the sample recognized that achieving cultural competence involves a commitment to learning over a lifetime.

An awareness that stereotypical attitudes and discriminatory actions can dehumanize and even encourage violence against individuals because of their membership in groups that are different from themselves was reported as high by the majority of the participants. Aligned to the previous satisfactory high percentages, participants respond that they know their family’s story of immigration and assimilation into Cyprus, and they continue to develop their capacity for assessing areas where there are gaps.

The second set of questions of the research tool evaluates the self-reported abilities of health professionals to manage diversity which is another pillar of cultural competence effectively.

In relation to the questions on health professionals’ skills, in response to the question of whether participants try to develop ways to interact respectfully and effectively with individuals and groups, 58.8% (n = 238) answered that they behave systematically well, while 37.5% (n = 152) often with a satisfying total of 96.6%.

When asked if they feel they can effectively intervene when they observe others behaving in a racist and/or discriminatory manner, the answers of the participants were varied. Fourteen point one per cent of the sample (n = 57), never intervene, 39.8% (n = 161) rarely intervene and only 46.2 (n = 187) intervene actively. It is worth noting that people who come from urban cities reported they intervene more often compared to people who come from the countryside, with a statistically significant difference of *p* = 0.03.

A high rate of 93.1% of participants (n = 377) answered that they could adapt their communication style to effectively communicate with people who communicate in ways that are different from theirs.

In response to the question: “I seek out people who challenge me to maintain and increase the cross-cultural skills I have,” responses included at all times or very often, 44% (n = 178) and 44.7% (n = 181), respectively.

To the question ‘I am actively involved in initiatives, small or big, that promote understanding among members of diverse communities.’ There were 27.9% (n = 113) of the participants that reported not being actively involved, while 41.2% (n = 167) reported sometimes, and 30.9% (n = 125) almost always.

In the question as to whether the participants can act in ways that demonstrate respect for the culture and beliefs of others, 78.3% (n = 317) replied always and 19.8% (n = 80) almost always.

In keeping with previous questions, another asked participants if they are learning about and putting into practice the specific cultural protocols and practices necessary for their work; 82.2% answered positively. People who had previously attended seminars about cultural issues answered positively, with a statistically significant difference compared with people who have not (*p* = 0.007).

When asked if their colleagues who are immigrants or colored consider them an ally and know that they will support them in culturally appropriate ways, if they work hard to understand the perspectives of others, and consult with diverse colleagues about culturally respectful and appropriate courses of action, the responses were positive. Participants who come from the countryside seem to better support their colleagues or friends compared to people who live in urban places, with a statistically significant difference of *p* = 0.023.

The last question asked if the participants know and use a variety of relationship-building skills to create connections with people who are different from them. Participants’ responses were positive in high percentages, too. Women seem to answer more positively compared to men (*p* = 0.008). Finally, the statistical significant differences can be found at Table 4.

#### 3.1.2. Findings from the Interviews

The findings from the interviews were analyzed with the content analysis method and grouped into two main categories; Beliefs and attitudes that reveal knowledge and experiences, which reveal their skills. Additional connections were made after taking into consideration data about participants’ gender, age, educational level, and origin. Although this was the qualitative part of the research, an indicative quantification took place, categorizing responses into positive or negative after a thorough discussion with the research team. From the semi-structured interviews conducted with health professionals, the most important finding that emerged was the discrepancy between quantitative and qualitative data. In other words, while many participants rated their cultural competence highly when asked, they seemed to express themselves with stereotypes and often-racist comments. For example, in the questions relating to their knowledge of diversity, in the question of why they believe that some women from other countries experience abusive behaviors in clinical settings, the answers given were indicative of P17 “They also sometimes cause them and it is expected by a doctor or nurse to give in. Not everyone can have the same degree of resistance” or according to P21 “They are used to another communication pattern that is more intense or others need a more dynamic way to understand what you are telling them.”

When asked to describe how they handle an incident that occurs due to religious beliefs, the answers given indicated a low level of education and knowledge. Indicatively, P11 stated that “I will contact my boss to tell me how he wants us to handle it,” and P32 stated, “We have not received any relevant training on these issues. In my opinion, however, we must respect the patient’s beliefs and ask the family what they want us to do”.

When we asked about the active involvement of the participants in the practical support of a patient in an abuse case, P12 stated, “It is not my responsibility to get in the middle of a bad moment.” P21 said, “I am afraid to be actively involved. I’m also not sure if I’ll be attacked personally because I have been involved. I would prefer to verbally urge them to comply, but I would not want to get involved with a physical presence”.

Finally, to another question regarding the ways of self-education in issues related to the subject, indicative answers given are P2 “When the hospital designs such programs, I always attend and learn a lot. The last time we were trained on the patients’ rights last year, the participation of the staff was very high”. In the same line, P36 stated, “There is no time for study beyond the basic urgent obligations for my work. Nevertheless, I would like to participate in programs organized by either the Ministry of Health or the hospital where I work”.

An indicative quantified description of the qualitative data is available (Table 5). Responses that showed increased levels of cultural competence were named “positive responses,” while the rest of the answers were categorized as negative, just for the need of this visualization.

## 4. Discussion

The quantitative results of this research showed that most health professionals self-assess positively and state that they respect diversity. Of course, there are respondents that do not feel confident communicating efficiently with the different ‘Other’ and they need extra training. Combined with the study demographics, the results showed that nurses and psychologists were reportedly more aware of cultural competence issues and it may be that more socially oriented sciences are better preparing healthcare professionals to manage diversity in the healthcare context. However, there is a gap between knowledge and skills where doctors seem to be superior to nurses in practical skills and a willingness to intervene in cases of racist behavior or problem-solving actively. Mistakes during communication occur, but what is important is how each health professional manages them. Much of the time, participants tended to attribute mistakes to the shortcomings of individual interpreters rather than being seen as indicative of a wider need for cross-cultural awareness and sensitivity. Therefore, as stated in the literature, health professionals need to be sensitive to social and cultural differences [15]. The fact that 55 participants answered that they do not make mistakes suggests a lack of awareness of mistakes, which is also related to empathy [16].

Regarding the question about the recognition of the participants’ lack of knowledge and their positive intention to learn more, the results point in the direction of other recent studies that come to similar results [17,18,19]. The answers given about the willingness of the participants to listen before asking questions or about the fact that individual differences are an important part of people’s identities were satisfactory. Recent research states that professional caring values exert a partial mediating effect on the relationship between compassion, cultural competence, and mental health balance [17].

The questions about recognizing the fact that there can be cultural differences over time were also satisfactory. However, their knowledge of history was largely incomplete among all specialties within the sample, something which is not mentioned in other recent studies about racism in health care [18].

An awareness that stereotypical attitudes and discriminatory actions can dehumanize and even encourage violence against individuals because of their membership in groups that are different from themselves was reported as high by the majority of the participants. Aligned to the previous satisfactory high percentages is the response that participants know their family’s story of immigration and assimilation into Cyprus and they continue to develop their capacity for assessing areas where there are gaps [17,18,20].

An important result was that when they observe others behaving in a racist and/or discriminatory manner, they do not intervene. Participants’ responses varied in all possible options, which is in complete agreement with similar studies in other countries [20,21]. Similar results are found in surveys conducted in western countries, while results from studies in low and middle-income countries showed greater active involvement.

Equally low skills are recognized by participants in relation to issues of activism and involvement in voluntary actions that promote an understanding of diversity. This finding aligns with recent studies [20].

Almost 20% of the study population answered that they did not put into practice the specific cultural protocols and practices which are necessary for their work. Research in other countries comes to the same conclusion on the grounds of lack of time and increased workload [22].

In the following questions about their intention to build strong relationships with a diverse community, the participants show that they evaluate themselves highly and state that they have acquired the cultural ability to a significant degree without facing any difficulties or problems. Other studies come to similar conclusions [23].

The results of the interviews showed that whilst many participants rated their cultural ability highly when asked to discuss these issues; they seemed to express themselves with stereotypes and often racist comments. This has not been reported in the literature for the last decade and is an important finding that needs to be further explored.

Words isolated from participants’ responses, such as “some provoke,” indicate the existence of stereotypes that have not changed over time and focus on understanding and justifying the perpetrator who behaves inappropriately. Answers such as “a doctor or nurse are expected to give in” show a tacit acceptance of racist behavior along the same lines.

In addition to using stereotypes, they also refer to phrases such as “They are used to another pattern of communication and need a more dynamic way to understand what you are telling them.” Often, the literature records the disclaimer or feeling of inadequacy that some health professionals may have. Such a confirmation emerges from the results of the present study with phrases such as “I will communicate with my boss” or “It is not my responsibility to get in the middle of a bad moment” [24].

Something that has not yet been reported in cultural competence research is related to the expression of personal opinion and the lack of an evidence-based approach to what is related to the effective and personalized care of patients belonging to diverse communities. An indicative phrase that confirms the above is “In my opinion, however, we must respect the patient’s beliefs and ask the family as well.”

Some more important results deal with the fact that participants who had previously attended seminars in the field of cultural studies were answering more positively compared to people who did not care for their continuous training. People who come from urban places and especially women are more sensitive to diversity issues.

Finally, the data of the current study suggests that training of staff comes only from central initiatives of the state and not from the personal initiative of each professional.

## 5. Conclusions

Participants identify the importance of their cultural ability both for their greater effectiveness in their workplace and in their personal life. They also realize that it is important for optimal personalized health care planning. In self-assessment of their knowledge of transcultural communication, they rated themselves very high but acknowledged cognitive deficiencies in the history of cultures, individual history, and limited knowledge, mainly in matters of internal diversity. In relation to their skills, participants reported that they do not easily intervene in cases where they observe racist behaviors, engage in activist actions in support of people who fall into the spectrum of otherness, and do not self-educate due to a lack of motivation and time. In the same subject areas, however, when qualitative data were collected through semi-structured interviews, there seems to be a mismatch compared with responses given during the completion of the self-assessment research tool. While participants reported that they had mastered cultural competence to a significant degree, in free case analysis, they seemed to have incomplete knowledge and low skills. In fact, they report that they expect their education from third parties and do not take the initiative for self-education. Finally, in their comments, they refer to stereotypical perceptions of specific communities and often justify racist incidents. It is, thus, clear that there is a significant need for continuous and specialized training programs.

The limitations of this study include the fact that a person’s cultural competence is a general concept difficult to measure with the consequent possibility of misinterpretations. In addition, from the qualitative data, there is a significant risk of deviations from the precise determination of health professionals’ cultural competence.

From this study emerges the need to support the acceptance, inclusion, and finally, the cultural competence of specific communities such as the community of people with disabilities, the LGBTQ community, refugees, etc. Moreover, there is a need for the design of educational programs for health professionals either by the governments or by Universities and health/social care institutions.

## Figures and Tables

**Table 1:** Internal Consistency Validity.

Thematic Units	Cultural Competence Self-Assessment Checklist	Cronbach’s Alpha	Number of Questions
1	Knowledge	0.714	10
2	Skills	0.895	10
	Total	0.804	20

**Table 2:** Demographic Characteristics.

Characteristics	N	%	Characteristics	n	%
**Sex**			**Cultural Studies Knowledge**		
**Men**	68	16.8	**yes**	161	39.8
**Women**	337	83.2	**no**	244	60.02
**Age (years)**			**Place of residence**		
**15–30**	148	36.5	**municipality**	196	48.4
**31–40**	175	43.2	**province**	209	51.6
**≥40**	82	20.2			
**Qualifications**			**Specialty**		
**BSc**	126	31.1	**Nurse**	218	53.8
**MSc**	278	68.6	**Doctor**	89	22
**PhD**	1	0.2	**Midwife**	38	9.4
			**Psychologist**	38	9.4
			**Other**	22	5.4

**Table 3:** Scale results.

Questions	Never/Not at All		Sometimes/Good		Often/Fairly Good		Always/Excellent	
	n	%	n	%	n	%	n	%
I make mistakes in my behavior in relation to people who are characterized by diversity and I learn from them	55	13.6	127	31.4	168	41.5	55	13.6
I realize that my knowledge of specific cultural groups is limited and I would like to know more	5	1.2	33	8.1	163	40.2	204	50.4
I am interested in listening well before moving on to the next questions when communicating with people who are different.	1	0.2	19	4.7	141	34.8	244	60.2
I know that differences in color, nationality, etc. are important elements of someone’s identity that have equal value and I do not hide it in my conversations with others	25	6.2	25	6.2	153	37.8	202	49.9
I know a lot about the history of people from Eastern or African countries as well as the history of minorities in my country	50	12.3	177	43.7	135	33.3	43	10.6
I recognize that cultures change depending on individuals and time	3	0.7	39	9.6	195	48.1	168	41.5
I understand that cultural competence means, at the same time, lifelong learning on issues of diversity	3	0.7	25	6.2	151	37.3	226	55.8
I recognize that stereotypes can encourage exclusion, violence, and injustice	3	0.7	7	1.7	90	22.2	305	75.3
I know my family history	8	2	22	5.4	134	33.1	241	59.5
I deal with potential gaps in my knowledge of diversity and try to fill them	6	1.5	54	13.3	183	45.2	162	40
I develop ways to interact effectively and respectfully with individuals and groups	1	0.2	14	3.5	152	37.5	238	58.8
I intervene effectively when I observe racist behavior	6	1.5	51	12.6	161	39.8	187	46.2
I can adapt my communication style according to the circumstances and communicate effectively	1	0.2	27	6.7	206	50.9	171	42.2
I am looking for opportunities to acquire more transcultural skills	2	0.5	44	10.9	181	44.7	178	44
I am actively involved in initiatives that promote understanding of different groups	14	3.5	99	24.4	167	41.2	125	30.9
I behave with respect for the culture and opinions of others	1	0.2	7	1.7	80	19.8	317	78.3
I learn specialized transcultural topics that are necessary for my job	6	1.5	66	16.3	166	41	167	41.2
My colleagues who are characterized by diversity see me as their ally because I support them	6	1.5	32	7.9	172	42.5	195	48.1
I try to understand the needs of others and respect them even if I disagree with them	1	0.2	7	1.7	124	30.6	273	67.4
I enjoy building relationships/friendships, building skills, and making connections with people different than me.	3	0.7	28	6.9	159	39.3	215	53.1

**Table 4:** Statistically Significant Differences.

Question	Sex	Residence	Previous Education	*p* Value
B2	X			$x_{3}^{2}=8.09,p=0.046$
B7	X		X	$x_{3}^{2}=14.99,p=0.006$
B9	X	X		$x_{3}^{2}=14.03,p=0.004$
C4	X		X	$x_{3}^{2}=13.18,p=0.008$
C2		X		$x_{3}^{2}=8.65,p=0.003$
C8		X		$x_{3}^{2}=9.21,p=0.023$
B5	X		X	$x_{3}^{2}=11.21,p=0.01$
C7	X		X	$x_{3}^{2}=11.66,p=0.007$

**Table 5:** Interview Results.

Type of Questions	Participants	Positive Responses	Negative Responses
**Knowledge**	30 Nurses	22	8
	19 Doctors	7	12
	6 Psychologists	5	1
	2 Midwives	1	1
	5 Other	4	1
**Skills**	30 Nurses	12	18
	19 Doctors	10	9
	6 Psychologists	4	2
	2 Midwives	2	0
	5 Other	2	3

## Data Availability

The data presented in this study are available on request from the corresponding author.
